# Incidence and Characteristics of Pediatric Patients with Acute Otitis Hospitalized in a Romanian Infectious Diseases Hospital

**DOI:** 10.3390/children11070832

**Published:** 2024-07-08

**Authors:** Vlad Ștefan Pleșca, Anca Streinu-Cercel, Oana Săndulescu, Anca Cristina Drăgănescu, Răzvan Hainăroșie, Anca Doina Pleșca

**Affiliations:** 1 Faculty of Medicine, Carol Davila University of Medicine and Pharmacy, 050474 Bucharest, Romania; 2 National Institute of Infectious Diseases “Prof. Dr. Matei Balș”, 021105 Bucharest, Romania; 3 Academy of Romanian Scientists (AOSR), 050045 Bucharest, Romania

**Keywords:** children, acute otitis, viral infections, hospitalization

## Abstract

Background: Otic involvement is common in children during acute infectious diseases, and is an important cause of morbidity and health service utilization. Methods: We performed a retrospective analysis of pediatric cases hospitalized in the largest infectious disease hospital in Romania between 2018 and 2023, with the aim of quantifying the incidence and impact of acute otitis (AO) according to pediatric age subgroups. Results: A total of 1118 cases diagnosed with AO were eligible and included in the analysis. Acute congestive otitis media was the most common subtype, identified in 53.3% of cases, followed by acute purulent otitis media (APOM) in 26.7% of children. The majority of AO cases (69.9%) were diagnosed in the pre-pandemic period (2018–2019), and infants (10.6%), toddlers (49.4%), and preschoolers (29.2%) were the most affected age groups. A viral infection associated with the AO episode was documented in 49.6% of cases. Influenza viruses were most commonly reported (20.5%), followed by SARS-CoV-2 (5.8%), and adenovirus (4.9%). A total of 38 cases of AO were identified in children with measles. In 15.9% of APOM cases, *Streptococcus pneumoniae* was isolated by culture from otic secretions. The duration of hospitalization was longer in children with APOM and acute otitis externa compared to the other subtypes of AO (*p* < 0.001). Conclusions: Our study highlights the importance of ENT (ear, nose, and throat) monitoring in children hospitalized for acute infectious diseases, as the majority of AO cases occur in the context of a viral infection. These findings emphasize the necessity for tailored assessment and intervention in suspected cases of AO, especially in young children under 5 years of age.

## 1. Introduction

Otic involvement is commonly observed in children during acute infectious disease and is an important cause of morbidity and healthcare service use [1]. Acute otitis (AO) occurs commonly during or after an upper respiratory tract infection [2], either as a result of direct viral action, inflammation, or poor drainage of secretions, or as a result of bacterial superinfection [3]. The occurrence of AO is greater in the pediatric population than in adults. This is attributed to a number of anatomical, immunological, and behavioral factors unique to the pediatric age group [4,5]. In total, over 80% of children experience at least one episode of AO, with 51% of cases reported in children under the age of five [1].

AO is also a significant contributor to antibiotic prescribing in the pediatric population, although a significant proportion of cases can and should be managed without antimicrobial treatment. Analgesic therapy is the preferred treatment option, while antibiotic treatment may be delayed in children two years of age and older, or in those with mild symptoms [6]. Thus, otic involvement should be carefully assessed by the otolaryngologist (ENT specialist) to reduce unnecessary antibiotic consumption in the pediatric age group.

The aim of this study was to identify the specific characteristics of pediatric patients with AO according to the type of otitis, their age, and the underlying etiology. These data, derived from actual clinical practice, will contribute to the global data on otic involvement in children in the context of acute infectious diseases.

## 2. Materials and Methods

We conducted a retrospective study of pediatric cases (under 18 years of age) hospitalized at the National Institute of Infectious Diseases “Prof. Dr. Matei Balș” (NIID) who were diagnosed with AO. The NIID, situated in Bucharest, is the largest hospital in Romania dedicated to the management and treatment of patients with infectious diseases. The hospital has six wards and an emergency room for the care of children of all ages.

All consecutive hospitalized cases with a primary or secondary diagnosis of AO from January 2018 to December 2023 were eligible to be included in the analysis. Patient identification was performed using the following ICD-10 diagnosis codes: H60, H62, H65, H66, and H67.

The inclusion criteria for the study were as follows:

- Patient being under 18 years of age;
- An ear condition as determined by an ENT examination;
- Complete medical records;
- Patient records, including ENT evaluations, reviewed by an ENT physician on the study team to confirm inclusion and to classify the type of otic involvement.

Cases without an ENT examination in the medical records or those with incomplete data were excluded from the analysis.

We classified AO cases into acute otitis externa (AOE), acute congestive otitis media (ACOM), acute otitis media with effusion (AOME), and acute purulent otitis media (APOM). Cases showing involvement of the external ear canal with inflammation and edema, without involvement of the tympanic membrane or middle ear, were classified as AOE. Cases were included in the ACOM group if the tympanic membrane was congested, with no accumulation of secretions in the middle ear. Cases were classified as AOME if secretions accumulated in the middle ear without any bulging or tympanic membrane involvement, while cases were classified as APOM if secretions accumulated in the middle ear with bulging or pulsatility of the tympanic membrane, or tympanic perforation and purulent secretions.

For each patient, we collected general data (age, sex), clinical data (signs and symptoms), laboratory data (blood cell count; inflammatory syndrome: fibrinogen, erythrocyte sedimentation rate (ESR), and C-reactive protein (CRP); and culture from ear swab), etiological context (viruses identified by RT-PCR or serology for Epstein–Barr virus, cytomegalovirus, varicella zoster virus, and measles), and length of hospitalization.

Patients were divided into five age groups: infants (0–12 months), toddlers (13–35 months), preschoolers (3–4 years), school children (5–13 years), and adolescents (14–18 years). Any values of laboratory parameters not in the normal range were considered low or high, as appropriate.

Statistical analysis was performed using IBM SPSS version 25 software (IBM Corp., Armonk, NY, USA). After checking the data distribution, continuous variables are reported either as medians, accompanied by the interquartile range (IQR), or as means and standard deviation. The comparison of these variables was performed using the Kruskal–Wallis (H) test. For categorical variables, data are presented as frequencies and percentages, and comparison was performed using the chi-square test. In addition, Cramer’s V (V) coefficient was used to assess effect size. The threshold for statistical significance was set at a level of *p* < 0.05.

## 3. Results

### 3.1. General Characteristics

**Figure 1:**
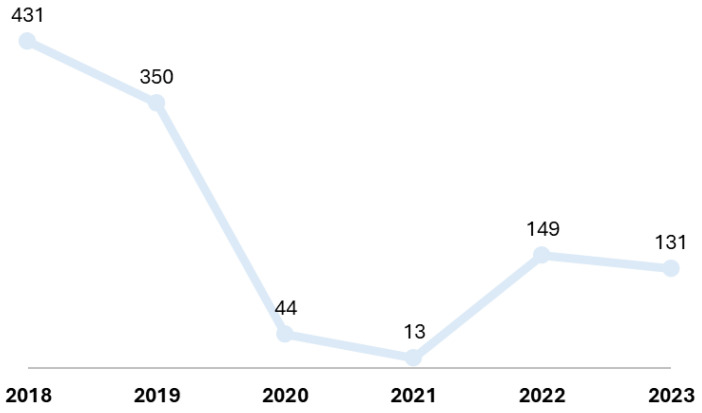
Distribution of cases by year.

A total of 1118 children were included in the analysis. Most cases were identified in the pre-pandemic years 2018 and 2019 (69.9%, *n* = 781, Figure 1). Male patients were more frequent (59.0%, *n* = 660), while almost half of the cases occurred in young children (49.4%, *n* = 552, Table 1), with a median age of 2.4 years (IQR: 1.5, 4.0 years).

### 3.2. Clinical and Laboratory Aspects in Relation to Age Group

The clinical picture was dominated by fever (88.7%, *n* = 992), cough (57.5%, *n* = 643), and nasal congestion (53.0%, *n* = 592). Among infants, the presence of nasal congestion was significantly associated with otic involvement compared to other age groups (*p* = 0.001, V = 0.126), while among school children and teenagers, headache (*p* < 0.001, V = 0.369) and otalgia (*p* < 0.001, V = 0.255) were significantly more common (Table 2).

Variations in white blood cell counts were observed between age groups (Table 2), and elevated serum CRP levels (74.4%) were more common than increased ESR (66.1%) or increased fibrinogen levels (32.4%).

The median duration from the onset of symptoms until hospital presentation was 4 days (IQR: 2, 6 days), with no significant variation between age groups (*p* > 0.05 for each).

### 3.3. Type of Otitis and Clinical and Laboratory Features

**Figure 2:**
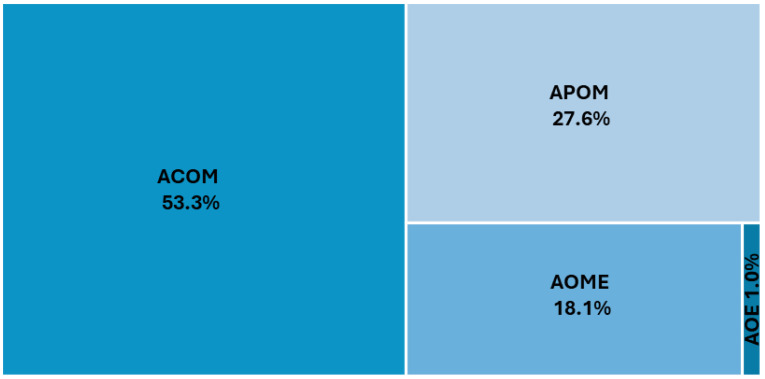
Distribution of otitis types in the study. AOE—acute otitis externa; ACOM—acute congestive otitis media; AOME—acute otitis media with effusion; APOM—acute purulent otitis media.

ACOM (53.3%, *n* = 596) was the most common type of otitis identified in patients in the study. Over a quarter of children (27.6%, *n* = 309) had typical APOM changes, and 18.1% (*n* = 202) were classified as having AOME. Only 11 (1.0%) children had AOE (Figure 2). The median age was significantly higher among children with AOE (4.5 years (IQR: 1.2, 7.3 years)) compared to the other types of AO (Table 3).

Otalgia was significantly more common in children with APOM (55.3%) and AOE (81.8%, *p* < 0.001, V = 0.185), while sore throat was reported more often in children with ACOM (29.9%) and AOME (28.7%, *p* < 0.001, V = 0.194).

APOM was associated with increases in white blood cells (*p* < 0.001, V = 0.185), neutrophils (*p* = 0.001, V = 0.120) and inflammatory markers such as fibrinogen (*p* = 0.002, V = 0.116), ESR (*p* = 0.019, V = 0.182), and CRP (*p* = 0.003, V = 0.159).

### 3.4. Type of Otitis and Clinical and Laboratory Features

In 555 cases (49.6%), a viral infection associated with the episode of AO was documented. Most commonly reported were influenza viruses (20.5%, *n* = 229), followed by SARS-CoV-2 (5.8%, *n* = 65) and adenovirus (4.9%, *n* = 55) (Table 4). A total of 38 cases of AO were identified in children with measles. AOE was only identified in the context of Epstein–Barr virus and varicella zoster virus infection. In 49 cases (15.9%, 49/309) of APOM, *Streptococcus pneumoniae* was isolated in the culture from otic secretion. In 31 of the 49 cases, tympanocentesis was performed, while in the other 18 cases, *Streptococcus pneumoniae* was identified from spontaneous otorrhea.

The length of hospitalization was significantly longer in children with AOE (6 days (IQR: 3, 8 days)) and APOM (6 days (IQR: 4, 7 days)) compared to ACOM (5 days (IQR: 4, 6 days)) and AOME (5 days (IQR: 4, 6 days)) (*p* < 0.001; H = 38.142). The length of hospitalization by age group and type of otitis did not vary significantly from these values for all patients (Table 5, *p* > 0.05 for each).

## 4. Discussion

This study identified that the majority of cases of otic involvement in children hospitalized for an acute infectious disease were ACOM (53.3%), followed by APOM (27.6%). These occurred mainly in infants, toddlers, and preschoolers. It is well established that AO is a frequent condition among children up to the age of five [7]. The concentration of a high number of cases in a relatively small population in terms of age range is not a mere coincidence. Rather, a number of anatomical, behavioral, and immunological factors predispose pediatric patients to the development of this condition. First, the anatomical features of the upper respiratory tract of children are directly implicated in the development of AO. The connection between the middle ear, which is an air-filled cavity, and the nasopharynx is via the Eustachian tube (ET), which regulates the drainage of secretions and pressure from the middle ear. The anatomy of the ET differs in infants and young children from that of adults. The shorter, narrower tube with a more horizontal pathway is more prone to inefficient drainage and provides easy access for pathogens, creating an environment that facilitates fluid stagnation and superinfection [8]. Second, the immaturity of children’s immune systems frequently results in their being affected by acute upper respiratory tract infections, which in turn often lead to otic complications [9]. Third, children’s hygiene measures are not always strictly observed, which serves to facilitate the spread of pathogens. Furthermore, breastfeeding in the supine position, the use of pacifiers, and exposure to passive smoking have also been identified as risk factors that can contribute to the development of acute otitis media.

We have shown that the number of cases of AO dropped dramatically during the COVID-19 pandemic, from 431 cases in 2018 and 350 cases in 2019 to 44 cases in 2020 and 13 cases in 2021. This significant reduction in the number of AO cases after the installation of lockdown measures was recently reported by Warner et al. [10] in a meta-analysis on studies from 11 countries. An overall decrease in all ENT pathologies was also reported during the COVID-19 pandemic [11]. It should also be noted that most of our cases in 2020–2021 were in the context of SARS-CoV-2 infection. This finding is not surprising given the decline in the circulation of other respiratory viruses in the first two years of the pandemic [12,13]. Ear pain, ear pressure, hearing loss, and dizziness have been reported in patients with COVID-19 [14]. SARS-CoV-2 has also been isolated in middle-ear fluid, showing direct involvement in the pathogenesis of AO [15]. In addition, during the COVID-19 pandemic, an increase in the incidence of AOE was reported [16]. In our analysis, we did not identify an increase in the number of AOE cases in the pediatric population associated with SARS-CoV-2 infection. The lower number of AO cases in the 2022–2023 period compared to the pre-pandemic period in our study may be artefactual, or due to the implementation of more widespread etiological testing through RT-PCR of pediatric cases hospitalized for influenza-like respiratory illness. Thus, etiology identification can warrant appropriate earlier management with a reduced likelihood of progression towards otic involvement.

The clinical picture dominated by fever (88.7%), cough (57.5%), and nasal congestion (53.0%) shows that most cases of AO occured in the context of viral upper respiratory tract infection. A study by Heikkinen and Chonmaitree [17] found that viral upper respiratory tract infections are involved in more than 70% of cases of childhood AO. Also, a cohort study conducted in Finland [18] showed that fever is a common symptom in children with acute otitis, being present in more than 80% of the cases studied, thus supporting the data obtained in our study. The identification and recognition of the typical symptoms of viral upper respiratory tract infections are crucial for the early diagnosis and effective management of acute otitis in children. It is imperative that all children hospitalized with upper respiratory tract symptoms undergo an evaluation by an ENT specialist. This specialist assessment allows for the early identification of signs of AO, thus facilitating the prompt initiation of appropriate treatment. In the context of hospitalization, the continuous monitoring of pediatric patients by ENT specialists can prevent the negative progression of the condition and ensure a personalized therapeutic approach tailored to the individual needs of each child.

In our study, we identified that, unsurprisingly, increased white blood cell counts, particularly increased neutrophil counts, along with increased ESR and CRP, are more commonly associated with APOM compared to other types of otitis. Evaluation by blood tests is essential in patients in whom a bacterial etiology of the otic involvement is suspected. Changes in white blood cell count may suggest viral or bacterial etiology, and inflammatory markers are useful in quantifying the body’s response to infection, as well as monitoring response to treatment.

In our analysis, 49.6% of cases exhibited a viral etiological context. In the development of acute otitis, influenza viruses were identified as the causative agent with the highest prevalence. The incidence of influenza in the pediatric population is high each season [19,20], and the risk of AO can increase 1.54 times during an influenza episode [21]. Furthermore, influenza vaccination in children has been shown to reduce the number of cases of AO in the pediatric population. Nevertheless, the rates of influenza vaccination, particularly among children, remain low in Romania. Recent data indicate that a relatively low percentage of the pediatric population is vaccinated against influenza each year [22]. To enhance influenza vaccination rates among children, it is imperative to implement efficacious information campaigns that underscore the safety and significance of vaccination. This is becoming increasingly important for Romania now that authorities ensure a better availability of vaccines and facilitate access to them through full reimbursement.

We identified a total of 38 cases of AO in children with measles. In the United States, AO is the most common complication of measles, with an incidence of more than 14% in children under 5 years of age, with lower rates seen with increasing age [23]. In addition, as with other viruses, rubella virus has been isolated from the middle-ear fluid of infants with acute otitis media [24], thereby demonstrating it can also have a direct involvement in the development of acute otitis media. Unfortunately, due to low measles, mumps, and rubella (MMR) vaccination rates in Romania [25], large measles epidemics have been ongoing in our country from 2016 to 2019 [25,26] and again since 2023, resulting in a high number of pediatric hospitalizations, many of which have been complicated by conditions beyond the field of AO.

Among the bacterial pathogens responsible for AO, notable species include *Streptococcus pneumoniae*, *Moraxella catarrhalis*, *Haemophilus influenzae*, *Staphylococcus aureus*, and *Streptococcus pyogenes*. Of these, pneumococcus is the predominant causative agent, accounting for more than 50% of cases of acute otitis, regardless of age [27,28]. This highlights the significant role of pneumococcus in the etiology of this condition, making it a critical target for both preventive and therapeutic strategies [29,30]. Non-typable *Haemophilus influenzae* is next in terms of isolation rate in cultures, being identified in up to 20% of cases occurring in children under six years of age [31]. *Moraxella catarrhalis* is detected in 10–15% of cases and is notably associated with recurrent infections due to its resistance to beta-lactam antibiotics, which are frequently employed as an empirical treatment for AO [32]. Also, group A streptococci, although not very frequently isolated in otic cultures, are responsible for cases occurring in older children, or those complicated with tympanic perforation or mastoiditis [33]. This spectrum of pathogens underscores the need for accurate diagnosis and targeted antibiotic therapy to effectively manage AO and mitigate complications. In our study, *Streptococcus pneumoniae* was isolated in 49 of the otic cultures from patients with APOM (31 by tympanocentesis and 18 by spontaneous otorrhea). Data on antimicrobial resistance or pneumococcal vaccination history were not available in this study due to its retrospective nature. The pneumococcal vaccine was widely introduced in the Romanian National Immunization Program for more than seven years, but nevertheless, more efforts are needed to ensure optimal vaccine uptake, and in the meantime invasive pneumococcal disease continues to remain a challenge in the country [34].

The main limitation of our study is the retrospective nature of the data, which made it impossible to fully monitor viral and bacterial etiologies. In addition, a proportion of the patient records had incomplete or brief ENT consultation data, or the type of AO was difficult to determine, so a proportion of cases had to be excluded from the analysis. Also, other data such as vaccine history or antimicrobial resistance of *Streptococcus pneumoniae* were not available for analysis. Nevertheless, the number of cases is high, and the 6-year duration of the study, including the pre-pandemic period, provides a comprehensive picture of the impact of AO on the pediatric population hospitalized in an infectious diseases hospital.

## 5. Conclusions

Our study highlights the importance of ENT monitoring in children hospitalized with acute infectious diseases, especially those presenting with fever, cough, and nasal congestion. The majority of AO cases were associated with viral infections, some of which were vaccine-preventable. Children under 5 years of age accounted for the majority of cases, and tympanic congestion was the most common finding. These data underscore the need for prompt evaluation and intervention in suspected cases of AO to prevent complications and reduce unwarranted antibiotic use.

## Figures and Tables

**Table 1:** General characteristics of the patients in the study.

Characteristics	Frequency, *n* (%)
Male sex	660 (59.0)
Infants	119 (10.6)
Toddlers	552 (49.4)
Preschoolers	327 (29.2)
School children	110 (9.8)
Teenagers	10 (0.9)
Chronic conditions (at least one)	54 (4.8)
Cardiovascular disease	9/54 (16.7)
Obesity	10/54 (18.5)
Diabetes	2/54 (3.7)
Leukemia	3/54 (5.6)
HIV infection	3/54 (5.6)
Hepatitis B virus infection	1/54 (1.9)
Neurological disease	12/54 (22.2)
Immunodeficiency (non-HIV)	4/54 (7.4)
Rheumatologic disease	2/54 (3.7)
Other chronic disease	11/54 (20.4)

**Table 2:** Clinical and laboratory characteristics by age group.

Characteristics	All Patients *n* (%)	Infants *n* (%)	Toddlers *n* (%)	Preschoolers *n* (%)	School Children *n* (%)	Teenagers *n* (%)	*p*-Value
	*N* = 1118	*N* = 119	*N* = 552	*N* = 327	*N* = 110	*N* = 10	
Clinical features							
Fever	992 (88.7)	106 (89.1)	486 (88.0)	296 (90.5)	96 (87.3)	8 (80.0)	0.678
Cough	643 (57.5)	77 (64.7)	325 (58.9)	180 (55.0)	55 (50.0)	6 (60.0)	0.176
Nasal congestion	592 (53.0)	75 (63.0)	307 (55.6)	164 (50.2)	42 (38.2)	4 (40.0)	0.001
Sore throat	213/999 (21.3)	NA	108 (19.6)	75 (22.9)	27 (24.5)	3 (30.0)	0.142
Headache	70/999 (7.0)	NA	10 (1.8)	23 (7.0)	33 (30.0)	4 (40.0)	<0.001
Otalgia	411/999 (41.1)	NA	105 (19.0)	210 (64.2)	89 (80.9)	7 (70.0)	<0.001
Malaise	111 (9.9)	5 (4.2)	64 (11.6)	29 (8.9)	10 (9.1)	3 (30.0)	0.026
Dyspnea	21 (1.9)	5 (4.2)	11 (2.0)	4 (1.2)	1 (0.9)	0 (0.0)	0.283
Laboratory parameters							
Increased WBC	472 (42.2)	46 (38.7)	240 (43.5)	136 (41.6)	49 (44.5)	1 (10.0)	0.286
Increased neutrophils	346 (30.9)	17 (14.3)	161 (29.2)	119 (36.4)	46 (41.8)	3 (30.0)	<0.001
Decreased neutrophils	98 (8.8)	22 (18.5)	46 (8.3)	20 (6.1)	9 (8.2)	1 (10.0)	0.002
Increased lymphocytes	50 (4.5)	23 (19.3)	21 (3.8)	4 (1.2)	2 (1.8)	0 (0.0)	<0.001
Decreased lymphocytes	169 (15.1)	5 (4.2)	54 (9.8)	62 (19.0)	40 (36.4)	8 (80.0)	<0.001
Increased monocytes	439 (39.3)	50 (40.2)	244 (44.2)	112 (34.3)	31 (28.2)	2 (20.0)	0.003
Increased fibrinogen	362 (32.4)	12 (10.1)	170 (30.8)	130 (39.8)	44 (40.0)	6 (60.0)	<0.001
Increased ESR	739 (66.1)	56 (47.1)	358 (64.9)	250 (76.5)	73 (66.4)	2 (20.0)	0.005
Increased CRP	832 (74.4)	70 (58.8)	393 (71.2)	270 (82.6)	89 (80.9)	10 (100)	0.003

NA—not applicable; WBC—white blood cells.

**Table 3:** Clinical and laboratory characteristics according to the type of acute otitis.

Characteristics	AOE *n* (%)	ACOM *n* (%)	AOME *n* (%)	APOM *n* (%)	*p*-Value
	*N* = 11	*N* = 596	*N* = 202	*N* = 309	
Age, years, median (IQR)	4.5 (1.2, 7.3)	2.3 (1.4, 3.9)	2.5 (1.7, 4.0)	2.4 (1.6, 4.1)	<0.001
Age groups					
Infants	2 (18.2)	73 (12.2)	17 (8.4)	27 (8.7)	<0.001
Toddlers	2 (18.2)	296 (49.7)	101 (50.0)	153 (49.5)	
Preschoolers	4 (36.4)	164 (27.5)	60 (29.7)	99 (32.0)	
School children	1 (9.1)	57 (9.6)	23 (11.4)	29 (9.4)	
Teenagers	2 (18.2)	6 (1.0)	1 (0.5)	1 (0.3)	
Clinical features					
Fever	8 (72.7)	526 (88.3)	184 (91.1)	274 (88.7)	0.253
Cough	3 (27.3)	355 (59.6)	115 (56.9)	170 (55.0)	0.114
Nasal congestion	1 (9.1)	242 (40.6)	72 (35.6)	110 (35.6)	0.077
Sore throat	2 (18.2)	178 (29.9)	58 (28.7)	34 (11.0)	<0.001
Headache	2 (18.2)	33 (5.5)	17 (8.4)	18 (5.8)	0.180
Otalgia	9 (81.8)	204 (34.2)	27 (13.4)	171 (55.3)	<0.001
Malaise	0 (0.0)	74 (12.4)	17 (8.4)	20 (6.5)	0.019
Dyspnea	0 (0.0)	12 (2.0)	4 (2.0)	5 (1.6)	0.941
Laboratory parameters					
Increased WBC	4 (36.4)	225 (37.8)	81 (40.1)	162 (52.4)	<0.001
Increased neutrophils	2 (18.2)	161 (27.0)	61 (30.2)	122 (39.5)	0.001
Decreased neutrophils	0 (0.0)	65 (10.9)	16 (7.9)	17 (5.5)	0.031
Increased lymphocytes	1 (9.1)	22 (3.7)	11 (5.4)	16 (5.2)	0.542
Decreased lymphocytes	3 (27.3)	91 (15.3)	30 (14.9)	45 (14.6)	0.724
Increased monocytes	4 (36.4)	228 (38.3)	73 (36.1)	134 (43.4)	0.320
Increased fibrinogen	1 (9.1)	179 (30.0)	57 (28.2)	125 (40.5)	0.002
Increased ESR	6 (54.5)	372 (62.4)	124 (61.4)	237 (76.7)	0.019
Increased CRP	11 (100)	431 (72.3)	128 (63.4)	262 (84.8)	0.003

AOE—acute otitis externa; ACOM—acute congestive otitis media; AOME—acute otitis media with effusion; APOM—acute purulent otitis media; WBC—white blood cells.

**Table 4:** Etiological context according to the type of otitis.

Viruses	All Cases *N* (%)	AOE *n* (%)	ACOM *n* (%)	AOME *n* (%)	APOM *n* (%)	*p*-Value
	*N* = 1118	*N* = 11	*N* = 596	*N* = 202	*N* = 309	
Influenza viruses	229 (20.5)	0 (0.0)	128 (21.5)	50 (24.8)	51 (16.5)	0.037
Respiratory syncytial virus	48 (4.3)	0 (0.0)	28 (4.7)	12 (5.9)	8 (2.6)	0.236
Rhinovirus	13 (1.2)	0 (0.0)	9 (1.5)	0 (0.0)	4 (1.3)	0.365
Adenovirus	55 (4.9)	0 (0.0)	24 (4.0)	15 (7.4)	16 (5.2)	0.227
Human metapneumovirus	3 (0.3)	0 (0.0)	2 (0.3)	1 (0.5)	0 (0.0)	0.717
Bocavirus	4 (0.4)	0 (0.0)	2 (0.3)	0 (0.0)	2 (0.6)	0.682
Human coronaviruses	27 (2.4)	0 (0.0)	14 (2.3)	9 (4.4)	4 (1.3)	0.567
Parainfluenza viruses	3 (0.3)	0 (0.0)	2 (0.3)	0 (0.0)	1 (0.3)	0.871
SARS-CoV-2	65 (5.8)	0 (0.0)	39 (6.5)	21 (10.4)	5 (1.6)	0.362
Epstein–Barr virus	41 (3.7)	2 (18.2)	20 (30.4)	9 (4.5)	10 (3.2)	0.215
Cytomegalovirus	7 (0.6)	0 (0.0)	5 (0.8)	1 (0.5)	1 (0.3)	0.798
Varicella zoster virus	22 (2.0)	3 (27.3)	11 (1.8)	4 (2.0)	4 (1.3)	<0.001
Measles	38 (3.4)	0 (0.0)	31 (5.2)	5 (2.5)	2 (0.6)	0.003

AOE—acute otitis externa; ACOM—acute congestive otitis media; AOME—acute otitis media with effusion; APOM—acute purulent otitis media.

**Table 5:** Median length of hospital stay (50th percentile) by age group and type of otitis.

	AOE	ACOM	AOME	APOM
	median (IQR) in days			
All patients	6 (3, 8)	5 (4, 6)	5 (4, 6)	6 (4, 7)
Infants	6 ± 0 *	5 (4, 7)	4 (3.5, 9)	6 (5, 8)
Toddlers	8 ± 2.8 *	5 (4, 6)	5 (4, 6)	6 (4, 8)
Preschoolers	4 ± 2.6 *	4 (3, 6)	4 (3, 6)	6 (4, 7)
School children	4 ± 0 *	5 (4, 6)	6 (5, 9)	6 (4, 7.5)
Teenagers	8.5 ± 4.5 *	7 (4, 9)	6 ± 0 *	7 ± 0 *

* Mean and standard deviation (mean ± SD) due to low number of cases. AOE—acute otitis externa; ACOM—acute congestive otitis media; AOME—acute otitis media with effusion; APOM—acute purulent otitis media.

## Data Availability

The data are available through reasonable request to the corresponding author. The data are not publicly available due to restrictions.
